# Otitis Media in a New Mouse Model for CHARGE Syndrome with a Deletion in the *Chd7* Gene

**DOI:** 10.1371/journal.pone.0034944

**Published:** 2012-04-23

**Authors:** Cong Tian, Heping Yu, Bin Yang, Fengchan Han, Ye Zheng, Cynthia F. Bartels, Deborah Schelling, James E. Arnold, Peter C. Scacheri, Qing Yin Zheng

**Affiliations:** 1 Department of Otolaryngology-Head and Neck Surgery, University Hospitals of Cleveland, Case Western Reserve University School of Medicine, Cleveland, Ohio, United States of America; 2 Department of Genetics and Genome Sciences, Case Western Reserve University School of Medicine, Cleveland, Ohio, United States of America; 3 Case Comprehensive Cancer Center, Cleveland, Ohio, United States of America; 4 The Transformative Otology and Neuroscience Center, Binzhou Medical University, Yantai, Shandong Province, China; Indiana University School of Medicine, United States of America

## Abstract

Otitis media is a middle ear disease common in children under three years old. Otitis media can occur in normal individuals with no other symptoms or syndromes, but it is often seen in individuals clinically diagnosed with genetic diseases such as CHARGE syndrome, a complex genetic disease caused by mutation in the *Chd7* gene and characterized by multiple birth defects. Although otitis media is common in human CHARGE syndrome patients, it has not been reported in mouse models of CHARGE syndrome. In this study, we report a mouse model with a spontaneous deletion mutation in the *Chd7* gene and with chronic otitis media of early onset age accompanied by hearing loss. These mice also exhibit morphological alteration in the Eustachian tubes, dysregulation of epithelial proliferation, and decreased density of middle ear cilia. Gene expression profiling revealed up-regulation of *Muc5ac*, *Muc5b* and *Tgf-β1* transcripts, the products of which are involved in mucin production and TGF pathway regulation. This is the first mouse model of CHARGE syndrome reported to show otitis media with effusion and it will be valuable for studying the etiology of otitis media and other symptoms in CHARGE syndrome.

## Introduction

Otitis media (OM), inflammation of the middle ear, is the most prevalent disease in children [1]. OM leads to 24 million pediatric office visits and $4 billion in health care costs annually in the United States [2]. The pathogenesis of otitis media is influenced by multiple factors such as exposure to infectious pathogens, Eustachian tube anatomy, immunologic status, genetic predisposition, and innate mucosal defense [3]. The observations of a higher incidence of OM in white infants than in black and Asian infants and of the high incidence of OM in patients with genetic disease, such as CHARGE syndrome studied in this paper, indicate that genetic factors play an important role in development of otitis media [4].

CHARGE syndrome, a multiple congenital anomaly syndrome, has an incidence of approximately 1 in 10,000 individuals globally [5]. CHARGE syndrome is characterized by retarded growth and development as well as abnormalities of the eye, heart, choana, genitalia, and middle and inner ear. [6]. Virtually all individuals with CHARGE syndrome have recurrent otitis media with effusion in their first year of life. Approximately two-thirds of individuals clinically diagnosed with CHARGE syndrome have a mutation in the *Chd7* gene [7], encoding the chromodomain helicase DNA-binding protein 7 (CHD7). Recent evidence suggests that *Chd7* functions as a regulator of genes which play a role in cell-lineage specification in the nucleus and as a positive regulator of rRNA biogenesis in the nucleolus [8], [9], [10].

Here we present a novel mouse model for CHARGE syndrome with a spontaneous deletion mutation in the *Chd7* gene. This mouse model exhibits a high incidence of otitis media and associated hearing loss together with several other features that are commonly observed in CHARGE patients such as eye abnormality, growth retardation, and balance defects. Otitis media was not reported in any of the previously described animal models for CHARGE syndrome and to date, there are no published studies that have examined the pathogenesis of otitis media in human CHARGE syndrome patients [11]. By further study, we found that Eustachian tube dysfunction, dysregulated epithelial proliferation, and decreased middle ear cilia density can explain the incidence of OM in *Chd7* mutant mice. This mouse model facilitates the study of serous otitis media etiology in CHARGE syndrome patients and helps establish a common pathway for development of otitis media in humans in general.

## Results

### Circling mice have a deletion mutation in the *Chd7* gene

We identified a spontaneous mouse mutant exhibiting head bobbing and circling behavior transmitted in an autosomal dominant fashion. To determine the chromosomal location of the *Ome* mutation, heterozygous *Ome* mice on the BALB/cByJ background were mated with inbred C57BL/6J mice to generate F1 mice. Those F1 mice that exhibited circling behavior were backcrossed to BALB/cByJ inbred mice to produce N2 mice. Ninety of 180 total N2 progeny were affected. From these 90, genomic DNA was prepared as previously described [12] and genotyped by polymorphic microsatellite marker analysis via agarose electrophoresis, utilizing standard conditions and protocols. The initial genome scan was carried out on pooled DNA samples. After detection of linkage on Chr 4, microsatellite markers *D4Mit149*, *D4Mit235*, *D4Mit181*, *D4Mit18*, *D4Mit1*, *D4Mit292*, and *D4Mit193* were scored on individual DNA samples. Candidate genes localized to the critical region between markers *D4Mit181* (4.23 cM, NCBI Build 37, 9501070–9501202) and *D4Mit18* (6.01 cM, NCBI Build 37, 13937604–13937829) were identified by scanning the Ensembl genome database (Release 62).

**Figure 1:**
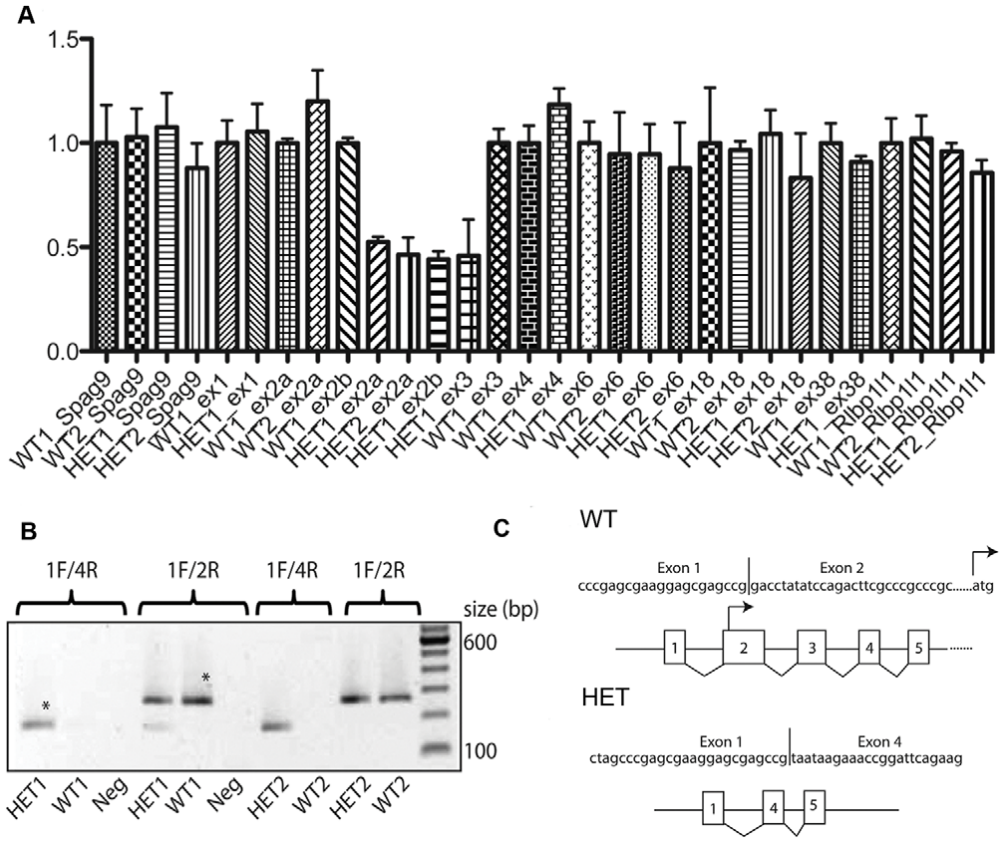
Identification of a heterozygous *Chd7* deletion mutation (*Chd7^Ome/+^*) in circling mice. (**A**) Quantitative PCR analysis of *Chd7* exons 1, 2, 3, 4, 6, 18, and 38 on chromosome 4 using genomic DNA isolated from wild-type (WT1 and WT2) and heterozygous mutant mice (HET1 and HET2). Genomic regions corresponding to the *Spag9* (on Chr 11) gene and the *Rlbp1l1* gene (also known as *Clvs1*; located on Chr 4, downstream of *Chd7*) were also amplified and serve as additional controls. Data were normalized to *Spag9* and the band for this gene was assigned the value of 1.0 (*y* axis). (**B**) qPCR analysis of cDNA generated from wild-type and *Chd7* mutant mouse brain RNA. PCR primers anchored in exons 1 and 4 (1F/4R) yield a positive result only for cDNA from heterozygous mutant mice. This product fails to amplify from wild-type cDNA because of its large size (∼2 kb). As expected for a heterozygous deletion mutation, PCR primers anchored in *Chd7* exons 1 and 2 (1F/2R) yield amplicons from both wild-type and mutant samples. “Neg” indicates a non-template control sample. A 100-bp standard was run in the lane near the right edge of the gel. Asterisks indicate bands that were isolated for sequencing in C. (**C**) Partial cDNA sequence of the bands isolated in panel B verifies that the mRNA transcribed from the mutant allele results from aberrant splicing of exons 1 and 4. The translation initiation codon is indicated in exon 2 of the wild-type allele, and has been deleted from the mutant allele.

Based on these mapping studies, and given that these mice show a remarkably similar phenotype to other mouse models with known mutations in *Chd7*, we hypothesized that these mice harbored a mutation in the *Chd7* gene, and set out to identify the causative mutation. Because most reported *Chd7* mutations are of the nonsense and frameshift type, we first sequenced the mouse *Chd7* gene by conventional Sanger sequencing to identify such a mutation. Several DNA variants were detected between *Ome* and mouse sequence NCBIM37; however, all were predicted to be benign polymorphisms (data not shown) and no difference between *Ome*/+ and littermate +/+. We then tested whether the mice harbored a heterozygous deletion or duplication mutation within the *Chd7* gene, which would have evaded detection by conventional DNA sequencing. To do this, we quantified *Chd7* exon copy number by quantitative PCR (qPCR) of genomic DNA isolated from mutant and wild-type mice. Relatively similar levels of *Chd7* exons 1, 4, 6, 18, and 38 were detected between wild-type and mutant mice (Figure 1A). However, *Chd7* exons 2 and 3 were amplified to levels approximately one-half of those in wild-type mice, consistent with a heterozygous deletion of these two exons. Genomic deletion of *Chd7* exons 2 and 3 resulted in an mRNA transcript with exon 1 aberrantly spliced to exon 4 (Figure 1B, C). The translation initiation codon is located in exon 2, and in silico translation of the aberrant *Chd7* transcript predicts a protein containing a premature stop codon. We therefore conclude that the deletion of *Chd7* exons 2–3 is the causative mutation in these mice, likely resulting in haploinsufficiency of *Chd7*.

### Ocular anomalies, growth retardation, and circling behavior in mutant mice

Keratoconjunctivitis sicca was observed in about 60% of the mutant mice (Table 1). Though mutant mice have smaller body size and weight at weaning age (Table 1), their life span is comparable to that of wild-type mice. Mutant breeder males can live at least 12 months until retired from breeding (data not shown). *Chd7^Ome/+^* male mice were slightly less fecund than wild type, but most breeding pairs succeeded to generate offspring. *Chd7^Ome/+^* mice also showed circling behavior (Video S1) and poor swimming ability (Video S2), which directly indicates a balance defect, compared to a normal swimmer (Video S3).

**Table 1:** Phenotype penetration in *Chd7^Ome/+^* mice.

Phenotype	Indicator	Mutant mice	Wild-type mice
Keratoconjunctivitis sicca	Gross examination	9/15 (60%)	2/16 (12.5%)
Mild growth retardation	Body mass at age P21 (mean)	10.3±2.7g*	12.4±2.0g
Hearing impairment	ABR threshold	10/10 (100%)	1/10 (10%)
Circling/ head bobbing	Gross examination	10/10 (100%)	0/10 (0%)

### Hearing deficiency was detected in *Chd7* mutant mice

**Figure 2:**
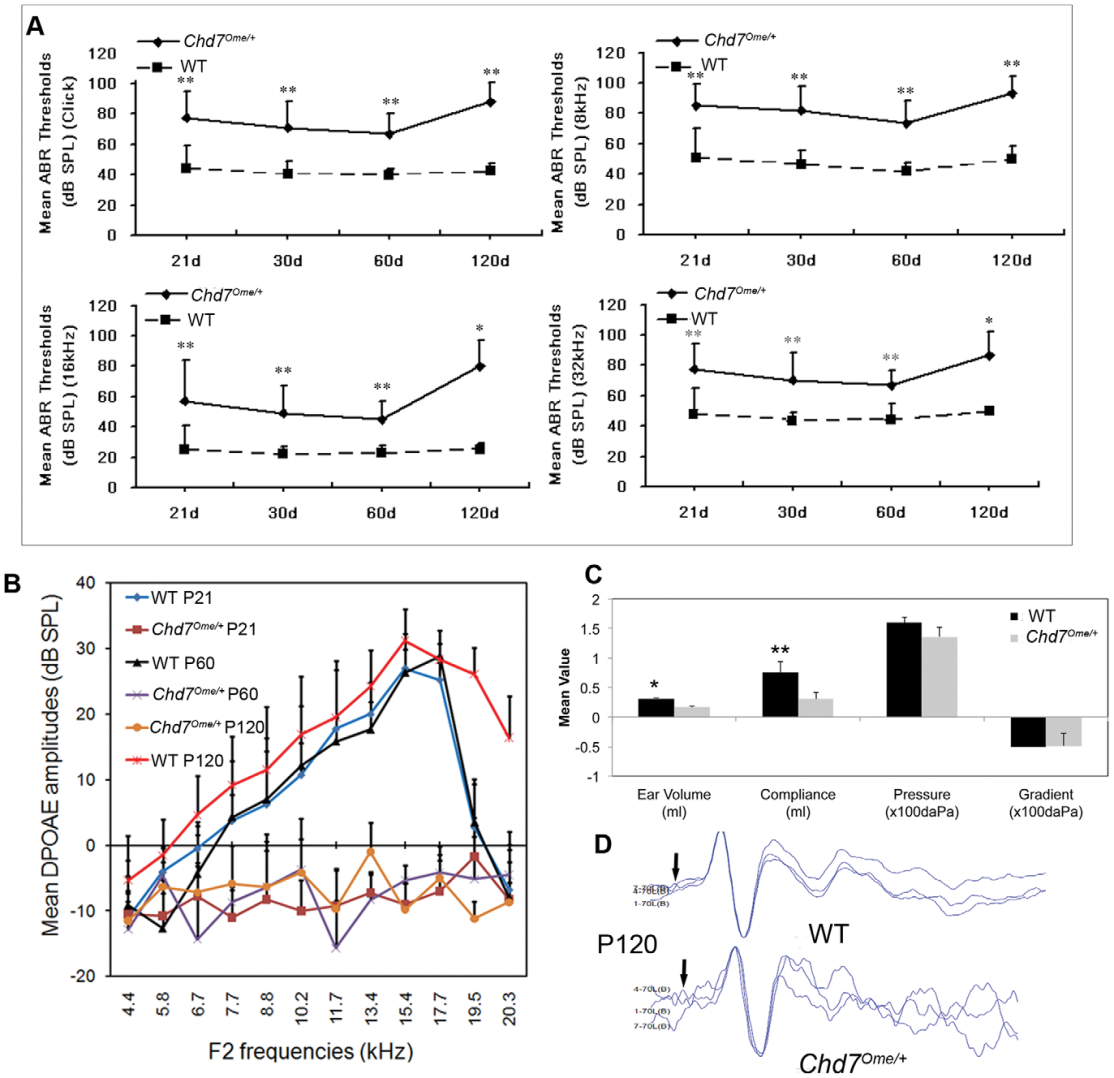
Evaluation of middle and inner ears in *Chd7* mutant and littermate control mice. (**A**) ABR thresholds in *Chd7^Ome/+^* and *Chd7^+/+^* littermate mice. *Chd7^Ome/+^* mice ranging in age from P21 to P120 (n = 13, 13, 7 and 3, respectively, in each age-group) and *Chd7* ^+/+^ littermate mice (n = 11, 17, 17 and 4, respectively, in each age-group) were assessed for ABR thresholds at click, 8 kHz, 16 kHz and 32 kHz. Mean ABR thresholds in *Chd7^Ome/+^* mice were about 20 dB SPL higher than mean thresholds of *Chd7^+/+^* littermate mice for all the frequencies tested, with significance indicated by asterisks (**P<0.01). (**B**) DPOAE amplitudes in a different set of mutant and wild-type mice at P21 (n = 4), P60 (n = 6) and P120 (n = 6). DPOAE amplitudes of mutant mice were undetectable at all three ages while control mice had normal DPOAE amplitudes. Error bars indicate standard deviation from the mean at each time point, for each mouse group. (**C**) Comparison of the values of tympanometry parameters in the right ears of *Chd7^Ome/+^* mice (n = 3) and littermate control mice (n = 4) at P90. Mean ear volume and compliance values in the mutant mice were significantly lower than those of littermate control mice (*, P<0.0001; **, P<0.05). (**D**) Both wild-type (WT) and *Chd7^Ome/+^* mice had normal CM recording, indicating normal inner ear function in the *Chd7* mutant mice even though DPOAE amplitudes were absent (data not shown). CM peaks from left ears of one mutant *Chd7^Ome/+^* mouse and one littermate control at 70 dB SPL are displayed.

A time-course observation of the ABR thresholds of *Chd7^Ome/+^* and wild-type mice is shown in Figure 2. The results showed that the mean ABR thresholds of *Chd7^Ome/+^* mice in each age-group from P21 to P120 (n≥3 at each time point) were significantly higher than those of wild-type mice at all the frequencies tested (Figure 2A). We also performed DPOAE tests over a similar time course and the results showed that mutant mice have amplitudes below zero at every frequency tested from P21 to P120 (n≥3 at each time point), substantially lower than those in wild-type mice (Figure 2B). Tympanometry measurement showed lower compliance values in the mutant mice (Figure 2C), which indicate abnormal middle ear function in the mutant mice. Though the mutant mice with otitis media tended to show cochlear microphonics (CM) responses at higher sound pressure levels compared to responses at lower sound pressure levels in littermate control mice, all the mutant mice (n = 5) did successfully generate CM peaks (Figure 2D).

### Otitis media was observed in a majority of *Chd7^Ome/+^* mice

**Figure 3:**
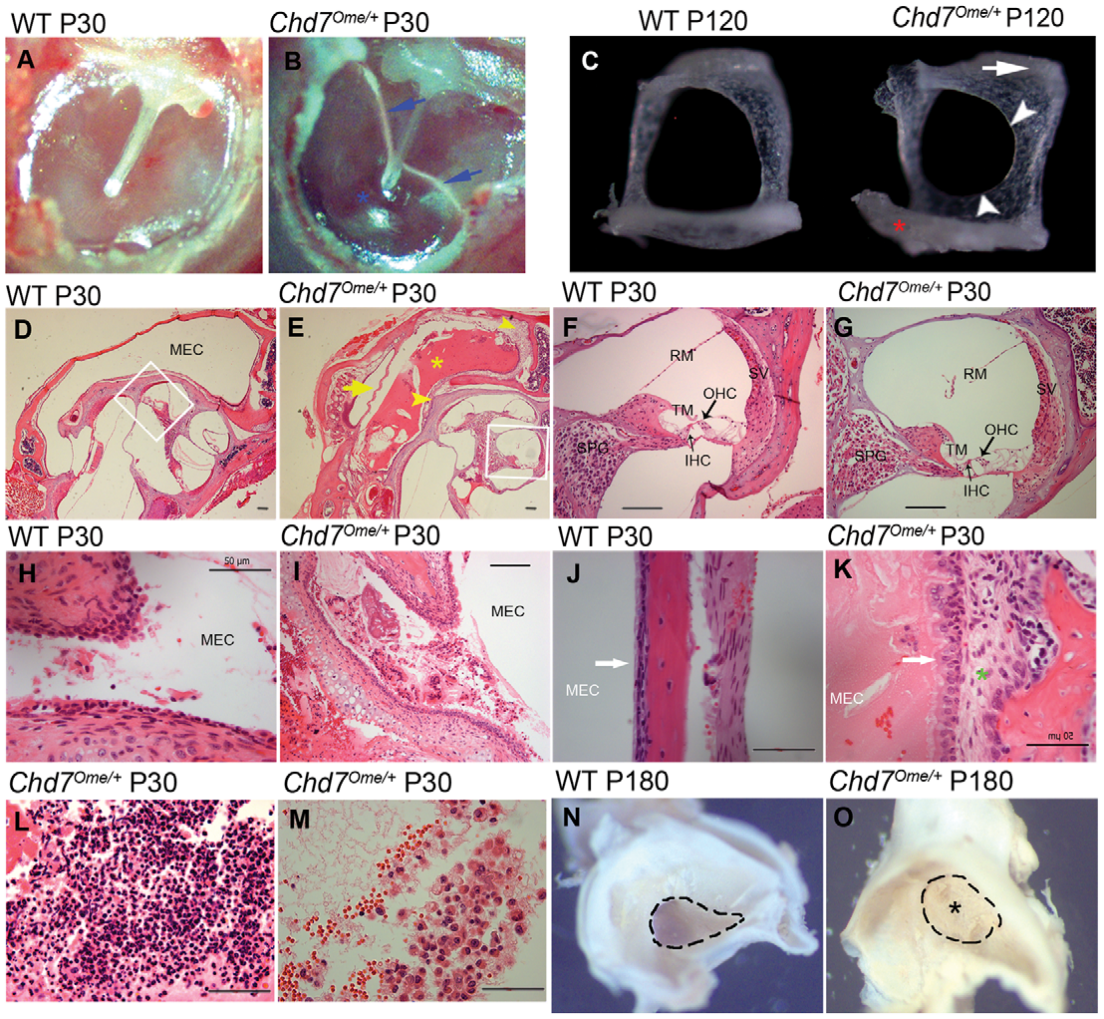
Middle ear and inner ear histology in *Chd7* ^Ome/+^ mice. (**A**–**B**) Effusion (blue arrows) and tympanic membrane retraction (blue asterisk) can be seen in the mutant mice. (**C**) In the mutant mice, the head of the stapes is moved toward the anterior crus (white arrow); the obturator foramen is smaller because of inward growth of the footplate and anterior crus (white arrowheads); the footplate is thinner and partially fused with surrounding bones (red asterisk). (**D**–**E**) In mutant mice, effusion was evident with a small amount of inflammatory cells (yellow asterisk); the tympanic membrane (yellow arrow), middle ear epithelia (upper right yellow arrowhead) and round window ridge (central yellow arrowhead) were thickened. (**F**–**G**) Enlarged images from corresponding control and mutant mice (white rectangles in D and E) to show the inner ear morphology; SV, stria vascularis; RM, Reissner's membrane; TM, tectorial membrane; IHC, inner hair cell; OHC, outer hair cell; SPG, spiral ganglion neuron. (**H**–**I**) Eustachian tube openings in the control and mutant mice. MEC, middle ear cavity. (**J**–**K**) Dilated periostea (green asterisk) and thickened epithelia (white arrows) were observed in the mutant mice. (**L**–**M**) Inflammatory cells in the MEC of mutant mice. (**N**–**O**) Eustachian tube openings in six-month-old mutant mice and littermate control mice. Scale bars: D, E, F, G, I = 100 µm; H, J, K, L, M = 50 µm.

**Figure 4:**
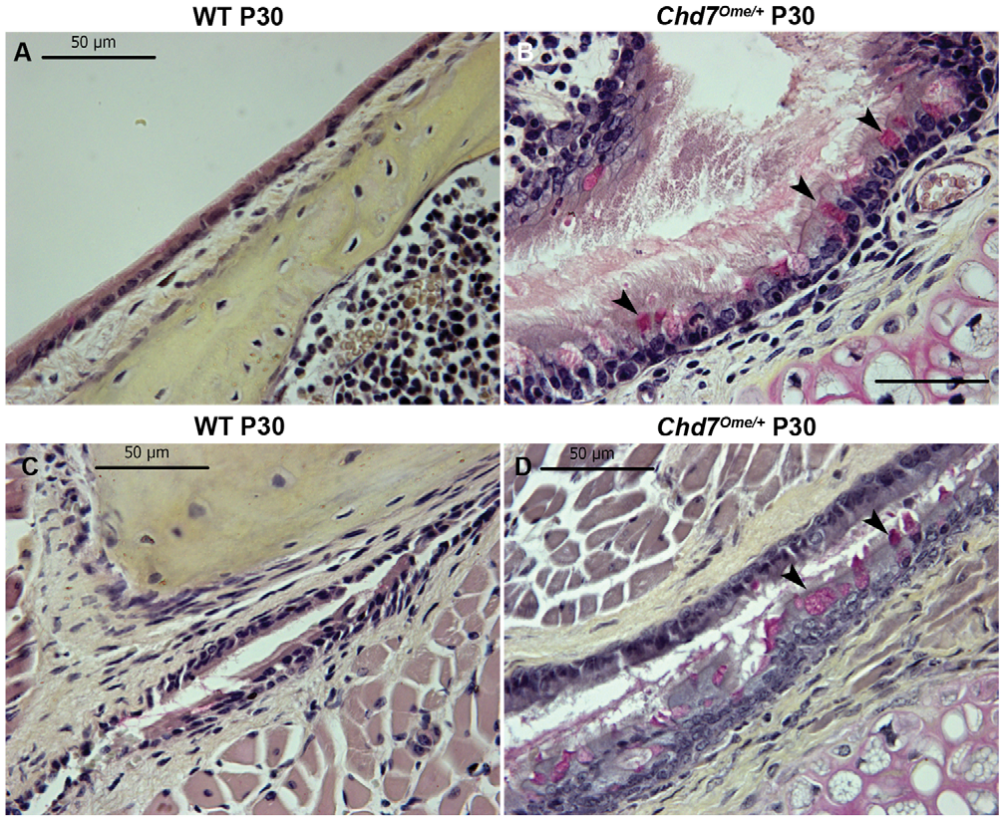
Representative sections of the middle ear and Eustachian tube mucosae of *Chd7* ^+/+^ and *Chd7^Ome/+^* littermates. Few goblet cells could be found in the middle ear (**A**) and Eustachian tube mucosae (**C**) of *Chd7^+/+^* littermate mice. By contrast, goblet cells were present at high density among other cells in the epithelium of the middle ear cavity (**B**) and the duct of the Eustachian tube (**D**) of *Chd7^Ome/+^* mice (typical goblet cells are indicated by arrows). Goblet cells have a distinctly polarized morphology in which the nucleus stains black in color at the cell base, and the mucus stains a deep rose color in the middle and apical portions of the cell. Scale bars  = 50 μm.

To evaluate the incidence rate of otitis media in *Chd7^Ome/+^* mice, 12 mice ranging in age from 21 to 77 days were randomly selected for otitis media screening by otoscopic and histological analysis. Of the 12 *Chd7^Ome/+^* mice examined, all 12 had effusion in the middle ear space of one or both ears, although the effusion volume varied (Table 2). Figure 3 shows representative images of the histology of *Chd7^Ome/+^* mouse ears. Effusion (Figure 3B, E, K), thickened epithelia (Figure 3E, K), a dilated periosteum (Figure 3K), and inflammatory cells (Figure 3L, M) were observed in most of the mutant mice. Effusion with inflammatory cells was also observed in the Eustachian tube opening in the middle ear cavity of most of the mutant mice (Figure 3I). The effusion content was quite variable with respect to quantity of inflammatory cells, which consisted mainly of polymorphonuclear cells (Figure 3L, M). Additionally, in *Chd7^Ome/+^* mice, goblet cells were found at higher density among other cells in the epithelium of the middle ear cavity (Figure 4B) and in the Eustachian tube duct (Figure 4D). By contrast, control mice exhibited few goblet cells in comparable sections of the mucosae of the middle ear cavity and Eustachian tube (Figure 4A and C). Hair cells and spiral ganglion cells were normal in both mutant and wild-type mice (Figure 3F, G). We also observed abnormal stapes in the mutant mice (Figure 3C). The head of the stapes in the mutant mouse was shifted toward the anterior crus and the obturator foramen was smaller because of inward growth of the footplate and anterior crus; the footplate was thinner and partially fused with surrounding bones. In studying the middle ear histology of P11 and P15 mice, we found that the onset of otitis media in the *Chd7* mutant mice can be as early as 11 days old (data not shown).

**Table 2:** Semi-quantitative evaluation of middle ear inflammation in *Chd7^Ome/+^* mice

Mouse	ID	Age	Effusion	Inflammatory cells	Tissue debris	Tissue proliferation
Wild type	1	**22d**	**–**	**–**	**–**	**–**
	2	**27d**	**–**	**–**	**–**	**+**
	3	**29d**	**–**	**–**	**–**	**–**
	4	**31d**	**–**	**–**	**–**	**–**
	5	**31d**	**–**	**–**	**–**	**–**
	6	**35d**	**–**	**–**	**–**	**+**
	7	**35d**	**+**	**+**	**–**	**+**
	8	**35d**	**+**	**–**	**–**	**+**
	9	**35d**	**+**	**+**	**+**	**+**
	10	**35d**	**–**	**–**	**–**	**–**
	11	**37d**	**–**	**–**	**–**	**–**
	12	**77d**	**–**	**–**	**–**	**–**
	13	**77d**	**–**	**–**	**–**	**–**
	14	**77d**	**–**	**–**	**–**	**–**
	15	**77d**	**–**	**–**	**–**	**–**
	16	**77d**	**+**	**–**	**–**	**–**
Mutant	1	**21d**	**+++**	**++**	**++**	**++**
	2	**22d**	**+++**	**+**	**+**	**+++**
	3	**22d**	**++**	**+**	**+**	**+++**
	4	**22d**	**+**	**+**	**+**	**++**
	5	**22d**	**+++**	**++**	**++**	**+++**
	6	**27d**	**+++**	**++**	**++**	**+++**
	7	**33d**	**+**	**+**	**+**	**+++**
	8	**37d**	**+**	**+**	**+**	**+++**
	9	**37d**	**+**	**+**	**+**	**+++**
	10	**41d**	**+++**	**++**	**+**	**++**
	11	**61d**	**+++**	**++**	**+**	**+++**
	12	**77d**	**++**	**++**	**–**	**++**

Mice from 21 to 77 days of age were used because of the early onset of otitis media in the mutant mice. Scoring system is described in the methods section. The total scored rate (85/144) of the four indicators in *Chd7* ^Ome/+^ mice was significantly higher than that (12/240) of the wild-type mice (P<0.01, chi-square test).

### No bacteria known to cause otitis media detected in the mutant or wild-type mice

To assess whether the otitis media with effusion in *Chd7^Ome/+^* mice is caused by bacteria, we applied PCR identification [13] and bacterial culture techniques to detect pathogens in the *Chd7^Ome/+^* mice and wild-type mice. Three specific primer pairs for the three most common otitis media-associated pathogens were used in a diagnostic PCR reaction [13]. None of the three pathogens (*Streptococcus pneumoniae*, nontypeable *Haemophilus influenzae*, and *Moraxella catarrhalis*) were detected (n = 4 for each group) by diagnostic PCR. Nor were the three pathogens detected in cultures, but comparable levels of *Streptococci* were identified in both mutant and wild-type mice (four mice of each genotype were analyzed).

### Decreased epithelial cilia density and increased goblet cell density in middle ears of mutant mice

**Figure 5:**
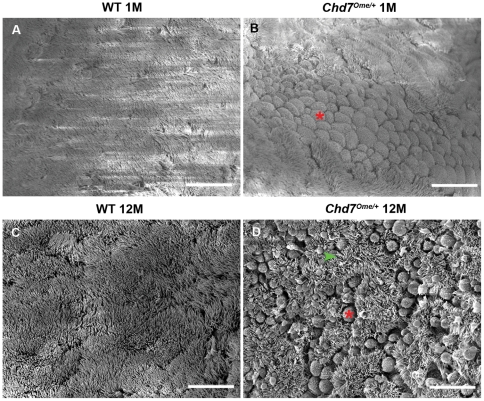
Scanning electron micrographs of middle ear cavities of *Chd7^Ome/+^* mice and littermate control mice. (**A, B**) Lateral views of Eustachian tube ostium epithelia in 1-month-old wild-type (**A**) and *Chd7^Ome/+^* (**B**) mice show increased goblet cells in *Chd7^Ome/+^* mice while ciliary morphology is indistinguishable from that in wild-type mice. Red asterisk identifies one of many goblet cells. (**C, D**) Rarefied cilia (green arrowhead) and scattered goblet cells (red asterisk) were observed in the mucociliary epithelia at the Eustachian tube ostia of 12-month-old *Chd7^Ome/+^* mice (**D**), while the cilia in age-matched wild-type mice (**C**) appeared normal. Scale bars  = 15 μm.

In the middle ear cavity, the Eustachian tube, and other organs including the lung, cilia function in clearance of normal secretions and abnormal fluids [14]. Patients with impaired ciliary function have increased incidence of otitis media [15]. Using scanning electron microscopy, we assessed the integrity of the mucociliary epithelium in 1-month-old and 12-month-old wild-type and *Chd7^Ome/+^* mice (n = 3 each genotype). At 1 month, the cilia in mutant mice were indistinguishable in number and morphology from those in wild-type mice, indicating that *Chd7* mutation had no effect on development of the middle ear cilia. However, increased goblet cells and swollen epithelia were observed in the mutant mice despite normal ciliary morphology (Figure 4B, Figure 5B). In 12-month-old mutant mice, rarefied and shortened cilia and a high density of goblet cells were observed in the epithelia (Figure 5D). These results indicate that progressive loss of cilia is associated with otitis media.

### Skull measurement reveals abnormalities in mutant mice

**Figure 6:**
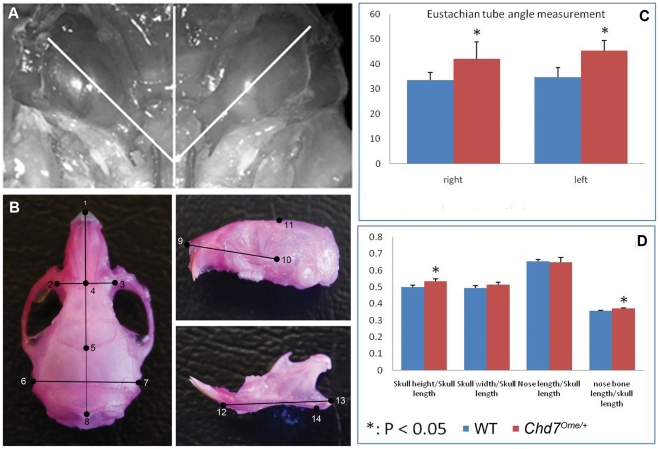
Skull and Eustachian tube measurement of *Chd7* mutant mice and littermate control mice. (**A, B**) Schematic diagrams superimposed on dissected skulls indicate the measured Eustachian tube angles (**A**) and skull dimensions (**B**). (**C, D**) Quantitative evaluation of the Eustachian tube angles and the skull dimensions in mutant and littermate control mice. Mutant mice have larger Eustachian tube angles of both ears than do the littermate control mice. Skull measurements indicated a greater skull height/skull length and nose bone length/skull length ratio in the mutant mice compared to littermate control mice.

Craniofacial abnormalities are observed in CHARGE syndrome patients and are often related to increased OM incidence; thus, skull measurements were performed on age-matched mutant and control mice to detect whether mutant mice differ in skull dimension. Mutant mice showed a larger skull height/skull length, larger nose bone length/skull length ratio and a greater Eustachian tube angle when compared to littermate control mice (Figure 6), indicating craniofacial abnormalities in *Chd7* mutant mice.

### *Chd7* postnatal expression and cytokine expression in the middle ear

**Figure 7:**
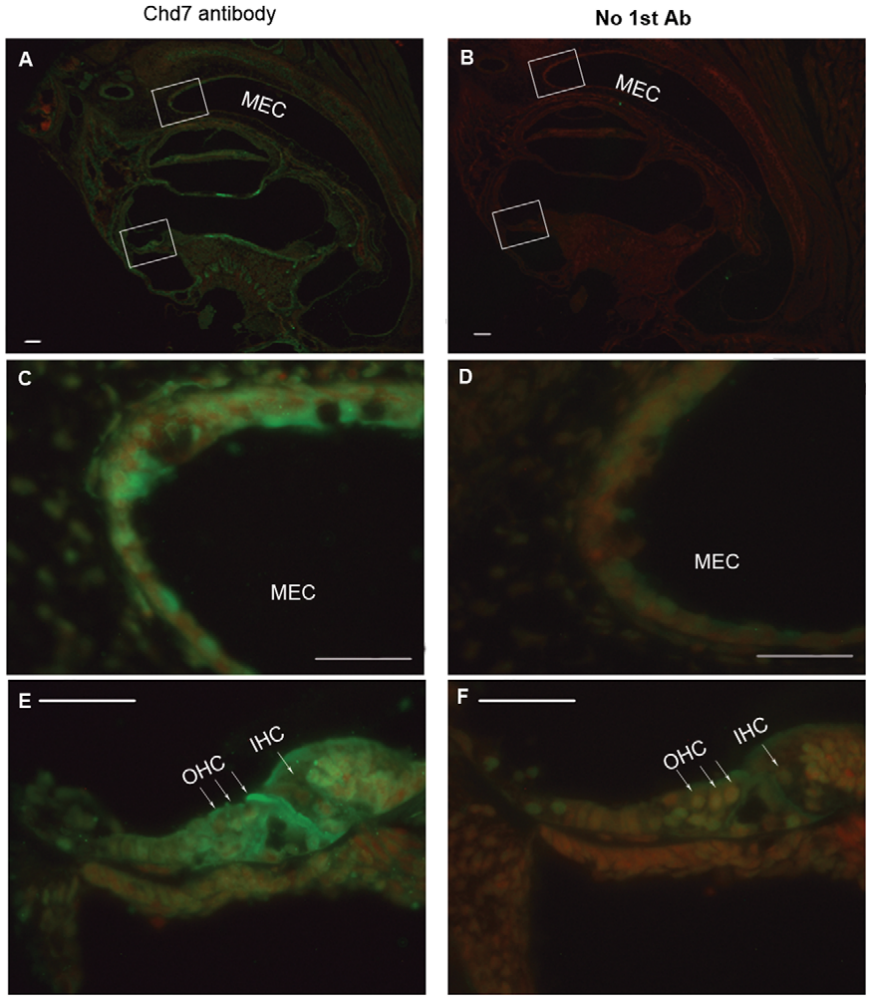
Immunohistochemical staining of *Chd7* protein in the developing middle ear. CHD7, revealed by green fluorescence, is expressed in the middle ear epithelium (**A, C**) and in the organ of Corti (**A, E**) in the inner ear of a wild-type mouse. MEC, middle ear cavity; IHC, inner hair cell; OHC, outer hair cell. Scale bars: A = 50 µm.

**Figure 8:**
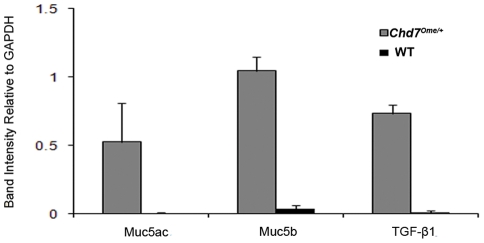
RT–PCR analysis of middle ears in *Chd7^Ome/+^* mice. Total RNA isolated from three *Chd7^Ome/+^* (mutant) and three wild-type littermate mice (control) were used to generate cDNA by reverse transcription. The cDNA was amplified using primers specific for *Gapdh*, *Muc5a*, *Muc5b* and *Tgf- β1*. The mutant mice showed significantly elevated expression levels for all three genes assayed, relative to *Gapdh* expression. Graph represents the digitized quantification of bands from an agarose gel. Error bars represent standard deviation from the mean, for each sample.

Studies have shown that *Chd7* is widely expressed during murine and human embryonic development [16], [17]. Recently, *Chd7* was found to be expressed in the developing human and mouse olfactory bulb and olfactory epithelium [18], [19]. To detect whether *Chd7* is expressed during development of the middle ear cavity and whether this expression may be related to the incidence of otitis media, postnatal wild-type mouse tissues were stained with anti- *Chd7* antibody. Immunohistochemical staining revealed expression of the *Chd7* protein in the middle ear epithelia and in the inner ear organ of Corti, limbus, and spiral ganglion cells (Figure 7A–E). We also investigated the expression levels of *Muc5a*, *Muc5b*, *Tgf-β1* and *Tlr2* genes which have been reported to be involved in the development of otitis media and inflammation. Total RNA was generated from middle ear tissue of equal mass from mutant and wild-type littermate control mice (all were 2 months old). cDNA was synthesized by reverse transcription for semi-quantitative RT-PCR to profile the gene expression levels. Our study showed elevated expression levels of *Muc5a*, *Muc5b* and *Tgf-β1* in the mutant middle ears compared to that in wild-type samples (Figure 8). *Chd7* (detected with primers *Chd7F* and *Chd7R*) and *Tlr2* (detected with primers *Tlr2F* and *Tlr2R*) genes were expressed in mouse ears, but transcription levels varied both in mutants and in wild-type littermate controls and did not show significant differences between the two genotypes (N = 8, data not shown).

## Discussion

Here we report the first spontaneous deletion mutation in the *Chd7* gene in mice. The phenotype and dominant inheritance of *Ome* mice are similar to those of mice with ENU mutagenesis-induced point mutations in the *Chd7* gene [19], including dizzy (*Dz*), eddy (*Edy*), and cyclone (*Cy*), and the unproven alleles wheels (*Whl*) and wheels-like (*Whll*). We report hearing loss in the *Chd7^Ome^* mutant mice and we provide evidence that otitis media is the major cause of hearing loss in this mouse model. The *Chd7^Ome/+^* mice also presented ocular abnormalities, balance defects, and growth retardation, all of which are consistent with the symptoms of human CHARGE syndrome patients and previously reported animal models for CHARGE syndrome [18], [19], [20], [21], [22], [23].

Hearing loss was observed in the *Chd7^Ome/+^* mutant mice starting from the age of weaning, and this loss was relatively stable over time. At younger ages (1–2 months old), the inner ears of the *Chd7* mutant mice appeared normal compared to littermate controls. There was very little hair cell loss; the density of spiral ganglion cells was comparable to that in the control mice, and no stereocilia loss was evident at this stage. However, in the middle ear, both effusion and thickened epithelia were observed in most of the mutant mice. Therefore, conductive hearing loss was dominant at this stage, which was also confirmed by the absence of DPOAE amplitudes in mutant mice. At an older age (7 months old), mutant mice observed by SEM exhibited hair-cell bundle loss from the cochlear basal turn, and this loss may have been caused by middle ear infection. At this stage, otitis media with effusion was predominant in the mutant mice; thus, mixed hearing loss caused by a combination of otitis media and stereocilia loss was dominant, in contrast to the earlier time point. Combining the results of histological and electrophysiological study, we conclude that hearing loss in the *Chd7* mutant mice is primarily caused by otitis media.

Otitis media is a multifactorial disease in which pathogenesis is affected by Eustachian tube structure and function, host immune status, innate mucosal defense, pathogen virulence, and strain and genetic susceptibility loci [3], [24]. Any genetic deficiency that influences one or more of these factors can promote susceptibility to otitis media. *Tlr2^-/-^* mice are more vulnerable to the challenge of gram-positive bacterium *S. pneumoniae* because of impaired bacterial clearance ability that leads to acute otitis media in this model [25]. *Eya4*-deficiency leads to Eustachian tube dysfunction and renders the mice susceptible to otitis media [2]. C3H/HeJ mice with a missense mutation in the *Tlr4* gene have a retarded response to endotoxin and an increased susceptibility to otitis media [26]. *Evi1*-null mice exhibited increased susceptibility to otitis media, caused by impaired transcriptional regulation of mucin genes in neutrophils through a TGF-β/SMAD signaling pathway [1]. Mutations in the *Fbxo11* gene discovered in *Jeff* mice lead to impaired regulation of TGF-β signaling and associated craniofacial abnormalities and thus cause impaired inflammatory responses in the middle ear [27], [28], [29]. In our study of *Chd7*-deficient mice, we found that abnormal Eustachian tube structure and function, dysregulated epithelial proliferation and decreased middle ear cilia density rendered these mice susceptible to otitis media with effusion.

The Eustachian tube performs critical functions affecting the middle ear in regulation of pressure, protection from nasopharyngeal secretions and other foreign material, and clearance of middle ear secretions [30]. Craniofacial abnormalities are often related to structural and functional changes in the Eustachian tube, thus increasing susceptibility to otitis media [29]. Our study shows that when compared to wild-type control mice, *Chd7* mutant mice have abnormal skulls and Eustachian tubes of altered shape and larger tube angle, indicating possible Eustachian tube dysfunction. Further histological analysis of the middle ear cavity from mutant mice revealed a narrow Eustachian tube lumen caused by epithelial proliferation and a high density of goblet cells which together lead to increased mucus. These features are evidence of impaired structure and function of the Eustachian tube in mutant mice, and such changes would mechanically impede clearance of secretions in the middle ear cavity, therefore rendering the mice more susceptible to recurrent otitis media.

In addition to impaired Eustachian tube function, mucosal hyperplasia is a major feature of otitis media, contributing to symptoms and causing irreversible middle ear disease [31]. Middle ears of the mutant and wild-type mice examined by H&E staining indicated that 100% of the mutant mice had moderate to severe epithelial hyperplasia with or without effusion from as early as P11, while middle ear cavities (MECs) of wild-type mice were clear and showed no abnormalities of MEC epithelial layers. Immunohistochemical staining of middle ears from young postnatal mice indicated postnatal *Chd7* expression in the MEC epithelia during middle ear cavitation. Early onset of otitis media confirmed that middle ear epithelia dysmorphology corresponded to developmental expression of *Chd7*. In the mucociliary (respiratory) type of epithelium of the middle ear cavity, there are numerous mucin-secreting cells [32]. Mucins secreted by these cells play an important role in several processes that are crucial for the protection and function of the underlying epithelium. These processes include mechanical protection of the epithelium, mucociliary clearance of pathogens and particulate matter, antigen presentation, and prevention of pathogen adherence and host invasion [32]. These mucins interact with pathogens and protect the underlying epithelium from invasion and mechanical damage.

Effusion was seen in most *Chd7^Ome/+^* ears but the amount and composition of the effusions varied with age. At early ages, most effusion was aqueous with very few inflammatory cells. Excessive effusion, even without inflammatory cells, can impede mucociliary clearance leading to mucostasis and associated hearing loss. Especially when epithelial proliferation is severe, impaired epithelial function and consequent diminished ciliary density make this process irreversible, thus causing chronic otitis media with effusion.

Immunodeficiency has been reported in CHARGE syndrome patients and is characterized by hypogammaglobulinemia, IgG2 subclass deficiency, impaired specific antibody responses, and T-B cell and/or natural killer cell defects [33], [34], [35], [36]. Bacterial culture indicated comparable bacteria levels in *Chd7* mutant mice and wild-type mice. Impaired immunity in the mutant mice could explain the infections in middle ears of mutant mice in the presence of bacteria flora.

In previously reported mouse models for otitis media, expression of the mucin gene family [37], [38] and the TGF signaling pathway [27] were affected. In this study, we profiled the expression levels of *Muc5ac* and *Muc5b* and *Tgf-β1* genes. Mucins are heavily glycosylated proteins that are considered primarily responsible for the gel-like characteristics of mucoid middle ear fluids. To date, 18 human mucin genes coding for mucin glycoproteins have been identified [39]. MUC5AC, MUC5B, and MUC2 are the key molecules that determine the properties of airway mucus gel [40]. These proteins participate in protecting the underlying middle ear epithelium from pathogen invasion and pathogen damage and assist with pathogen clearance. However, overproduction of these mucins can lead to increased viscosity of middle ear fluids, limited mucociliary clearance, and eventual mucin accumulation in the middle ear, ultimately leading to hearing loss. TGF-β1 is an important immunoregulatory mediator and participates in the middle ear inflammatory response and in other processes including apoptosis and tissue proliferation. Our discovery that mucin genes and TGF-β1 in the middle ear are upregulated is consistent with findings in human chronic otitis media patients [37], [38], [41]. These findings further support the *Chd7^Ome/+^* mutant mice as an ideal model to study the genetic pathways involved in human chronic otitis media. *Tlr2* gene expression was not significantly different between *Chd7^Ome/+^* mutant and control mice, suggesting that *Tlr2* is less involved in this chronic OM process. This result is consistent with the fact that no pathogenic bacteria were identified in this mutant though comparable levels of *Streptococci* were identified in both mutant and wild-type mice. In contrast, the TLR2-mediated immune response is significantly involved in *S. pneumoniae*-induced acute otitis media [25]. *Chd7* was expressed in mouse ears, which suggests that *Chd7* plays a direct role in ear development and ear disease processes including OM. *Chd7* transcript levels were not found to be significantly different between wildtype and *Chd7^Ome/+^* mutant mice, suggesting that the presence of the deletion mutation does not necessarily lead to non-sense mediated mRNA decay. However, due to a premature stop codon in the mutant transcript, the CHD7 protein level in heterozygous mice is predicted to be half that of the +/+ mice, and this haploinsufficiency of *Chd7* would lead to otitis media and other phenotypes.

*Chd7^Ome/+^* mice are a valuable model for human CHARGE syndrome because of a series of phenotypes that mimic human symptoms, including growth retardation, balance defects, otitis media, and hearing loss. Otitis media in *Chd7^Ome/+^* mice is characterized by Eustachian tube dysfunction, epithelial hyperplasia, middle ear effusion and associated hearing loss. All of these typical otitis media features make *Chd7^Ome/+^* mice a useful model for human otitis media. Further study of *Chd7*-associated genes that influence morphology and physiology of middle ear epithelia and the Eustachian tube may indicate new pathways with therapeutic potential for CHARGE syndrome and human otitis media.

## Materials and Methods

### Mice

Mice exhibiting head bobbling/circling behavior and with otitis media and eye defects (Ome mice) were first observed in at The Jackson Laboratory (JAX, Bar Harbor, Maine), then imported to Case Western Reserve University. Mating of an affected *Chd7^Ome^* ^/+^ male to a BALB/cByJ female revealed an autosomal dominant mode of inheritance. The mutant from a BALB/cByJ X CAST/Ei hybrid was backrossed 10 times to the BALB/cByJ background. Because circling females were not good breeders, mating pairs were usually arranged with a heterozygous male and a wild-type female. Mice were housed in ventilated racks at 21°C in a 12 h light–dark cycle with food and water given *ad libitum*. All animal protocols (R01DC009246, R01DC007392 and R21DC005846) were approved by the Institutional Animal Care and Use Committee of Case Western Reserve University.

### Sequencing and Genotyping

We sequenced genomic DNA, amplifying each exon of all the 40 exons of the *Chd7* gene (or two exons when they were near each other) separately by standard PCR, then performed Sanger sequencing using BigDye Terminator v3.1 Cycle Sequencing chemistry (ABI) and gave the reactions to the sequencing core for electrophoresis on an ABI 3730 DNA Analyzer. Primer pairs (data not shown) flanking each exon are designed according mouse genomic sequence (NCBIM37) Chromosome 4: 8,617,553–8,794,806 forward strand (Chd7 ENSMUSG00000041235). No DNA sequence difference was detected between Ome/+ and +/+ mice. Then, we performed the real-time PCRs with genomic and cDNA (using SYBR green PCR mix (Applied Biosystems) in an Applied Biosystems 7300 Real-Time PCR system) to compare quantitive DNA differences between Ome/+ and +/+ mice. Then we sequenced cDNA only to show that exon 1 was spliced to exon 4.

Because the precise location of the mutation is unknown, RT-PCR was used to genotype the mice. Primers anchored in exons 1 and 4 yield a 150-bp band for cDNA made from the heterozygous mice. These primers do not amplify a detectable product from wild-type cDNA under standard PCR conditions because of the product's large size (∼2 kb). Briefly, total RNA was prepared from the whole ear using the PureLink^TM^ Micro-to-Midi^TM^ Total RNA Purification System (Invitrogen). From each sample, 2 μg of total RNA template were used to synthesize cDNA using a SuperScript First-Strand Synthesis System. 1 μl of cDNA from each sample was subjected to PCR amplification in a Bio-Rad PTC-200 Peltier thermal cycler. Heterozygous mice were genotyped by detecting a truncated allele using a forward primer (1F, 5′-ctgactagcccgagcgaaggag-3′) and a reverse primer (4R, 5′-tggacacctgggtcttcgtc-3′) to yield a ∼150-bp band (Fig. 1). A second reverse primer (2R, 5′-cagactcccatcctcgccaaaaa-3′) anchored in exon 2 was used as positive control for the wild-type allele. Amplification conditions were 94 °C for 2 min; followed by 28 cycles of 94 °C for 30 s, 60 °C for 40 s and 72 °C for 50 s; followed by 5 min at 72 °C. Ten μl of the PCR products were subject to 2% agarose gel electrophoresis.

### Comprehensive evaluation of hearing loss in *Chd7* mutant mice

Hearing was evaluated in anesthetized (Avertin, 0.5 mg/g mouse mass) wild-type and *Chd7^Ome/+^* mice by using auditory brainstem response (ABR, measures auditory nerve and brainstem pathways involved in hearing), distortion product otoacoustic emissions (DPOAE, measures outer hair-cell function and predicts conductive hearing loss [42]), and tympanometry (measures tympanic membrane and ossicle chain function). A computer-aided evoked potential system (Intelligent Hearing Systems) was used to measure mouse ABR thresholds and DPOAE amplitudes as previously described [42], [43]. An Race Car tympanometer from Maico Diagnostics (Eden Prairie, MN, USA) was used to examine the tympaniogram following the procedures described previously [44].

**Table 3:** Sequences of primers used for RNA analysis.

Primer	Oligonucleotide sequence (5′–3′)
GapdhF	AACTTTGGCATTGTGGAAGG
GapdhR	GGAGACAACCTGGTCCTCAG
Muc5aF	TGGAAGGATGCTATCCCAAG
Muc5aR	CACCAGCATTGTGGGTACAG
Muc5bF	GACACCATCTATGGGGTTGG
Muc5bR	CAGGACTGTTCACCCAGGTT
TGF-β1F	AGCCCGAAGCGGACTACTAT
TGF-β1R	TCCACATGTTGCTCCACACT
Tlr2F	GAGCGAGCTGGGTAAAGTAGAAA
Tlr2R	AGCCGAGGCAAGAACAAAGA
Chd7F	AGACGCTCCTAAAAACAAGGACT
Chd7R	AACCCTTTCTTCTCCTGTCAAAG

### Histological analysis of middle and inner ears

Histological analyses of the middle and inner ears were performed following the methods described previously [45]. Briefly, middle and inner ears from *Chd7^Ome/+^* mice and wild-type mice were dissected after transcardial perfusion with 4% paraformaldehyde (PFA). Ear samples were immersed in 4% PFA for 48 h at 4°C, decalcified with 10% EDTA solution and embedded in paraffin. Sections (7 µm) were cut using an American Optical model 820 rotary microtome (American Optical, Buffalo, New York, USA), mounted onto Fisher Superfrost Plus slides (Fisher Scientific, Pittsburgh, PA) and counterstained in hematoxylin/eosin (H&E). Cryosections were prepared similarly but without dehydration, and instead, rinsed in a series of sucrose solutions and then embedded in Tissue-Tek OCT (Sakyra Finetek, Torrence, CA). Sections (10 µm) were cut with a Leica CM1900 cryostat microtome (Leica, Nussloch, Germany). Goblet cells, whose sole function is to secrete mucus, were identified by Mayer's Mucicarmine staining method following the protocol provided by Electron Microscopy Sciences (Catalog #26320).

### Scoring system for pathology of middle ears

A scoring system of -/+/++/+++ was used to assess the severity of pathology in the middle and inner ears following a previously described method [25], [45]. Histological analysis of the middle ears of control and mutant mice was performed using a light microscope (Leica DFC500, Germany). For the absence of pathology, a ‘–’ symbol was assigned. When pathology was very scarce in the middle or inner ear, a ‘+’ symbol was assigned. If pathology was more prevalent, but not to the point of spanning the entire middle or inner ear, then ‘++’ was assigned. If pathology spanned the entire middle or inner ear, then ‘+++’ was assigned. The pathologies being scored included middle ear effusion, inflammatory cell infiltration, tissue debris, and tissue proliferation. Both ears were examined in each mouse and only the one with more severe alteration was evaluated. Numerical scores were assigned to allow for semi-quantitative analysis of pathology (scoring 1 point for each +; for a total maximum possible score of 12 per mouse). A chi-square test was used to statistically evaluate the semi-quantitative data.

### Scanning electron microscopy **(**SEM**)**

Middle and inner ears from *Chd7^Ome/+^* and wild-type mice were dissected after transcardial perfusion with 4% paraformaldehyde (PFA) and then immersed in 2.5% glutaraldehyde in 0.1 M phosphate buffered saline (PBS, pH = 7.2) for 4 hours at 4°C. Dissection was performed to expose the middle ear cavities and inner ear basilar membrane. After post-fixation in 1% OsO4 in 0.1 M PBS (pH = 7.2, 1 hour), samples were washed in 0.1 M PBS (pH = 7.2), dehydrated in increasing concentrations of ethanol, dried in a BAL-TEC CPD 030 critical point dryer (BAL-TEC GmbH, Witten, Germany) according to manufacturer's instructions and analyzed in a Hitachi S-4500 scanning electron microscope (Hitachi, Tokyo, Japan) at 5 kV.

### Bacterial culture and identification

Bacterial culture and identification were performed following the protocol described previously [45]. Briefly, the bullae were removed and the middle ears were isolated under sterile conditions after ABR thresholds were determined. After microscopic examination, middle ears were washed with sterile PBS. 100 μl of the PBS lavage were inoculated onto a BBLTM Trypticase^TM^ Soy Agar plate with 5% sheep blood (TSA II, Fisher, Pittsburgh, PA, USA) and the plates were incubated at 37°C with 5% CO_2_ for 18 h. Bacterial colonies were sorted by colony appearance, counted and subjected to further identification.

### Immunohistochemistry

Cryosections were fixed in 1.5% PFA for 10 min. After being washed twice in 1x PBS and blocked in 5% bovine serum albumin for 1 h at room temperature, the samples were immersed in rabbit anti-*Chd7* polyclonal antibody (1∶200 dilution, Abcam, Inc., ab31824, Cambridge, MA) and incubated at 4°C overnight. The samples were washed twice in 1x PBS for 5 min each, immersed in goat anti-rabbit secondary antibody conjugated to Alexa Fluor 488 (1∶500 dilution; Invitrogen) and incubated at room temperature for 1 h. After counterstaining with PI for 15 min, the samples were mounted with Vectashield mounting medium (H-1000, Vector Laboratories). The samples were observed under an immunofluorescence microscope (Leica) and analyzed with Photoshop CS4 software.

### Preparation of the skull and craniofacial measurement

To measure craniofacial dimensions in control and mutant mice, the skulls were dissected and removed from 5 mutant mice and 8 control mice at age 3 months. Skulls were macerated in 1% potassium hydroxide overnight to remove any soft tissue and then stained with alizarin red, as previously described [46]. After staining, the skulls were stored in undiluted glycerin until measurements were taken. To measure the dimensions of interest, a hand-held digital caliper (General Tools & Instruments, NY) with a resolution of 0.01 mm was used. These instruments provide a precise and accurate mode of measurement for small distances, necessary here because great precision and accuracy is needed to distinguish differences between control and mutant mice. Measurements were recorded in millimeters and results for control and mutant mice were graphed; this method has been previously validated for accuracy and precision [47], [48]. Forty-four landmarks were adapted mostly from Dr. Joan Richtsmeier's paper on craniofacial dysmorphology in a Down syndrome mouse model [48], and from the standard measurement protocol in the Jackson Laboratory Craniofacial mutant source (Jackson Laboratory, Bar Harbor, ME http://craniofacial.jax.org/standard_protocols.html). Another measurement recorded was the angle between the midline of the skull base and the bony part of the Eustachian tube for both control and mutant mice. The skulls were then examined under an anatomical microscope and the skull base was photographed. The screen ruler software MB-Ruler 4.0 (Markus Bader, http://www.markus-bader.de/MB-Ruler/download.htm) was used to precisely locate the lines to be used for angle measurement and to measure the angles. The measurement data was statistically analyzed using the Student *t* test.

### RNA analysis

The same cDNA template and PCR conditions from the previous genotyping step were used to profile mRNA accumulation of *Muc5ac*, *Muc5b* and *Tgf-β1* in the *Chd7* mice by methods previously described [49]. PCR products were subjected to electrophoresis and the gray intensity of each band on a 2% agarose gel was digitized using ImageJ software (NIH) and corrected by the coefficient of *Gapdh* (glyceraldehyde-3-phosphate dehydrogenase) gene expression level of the same sample. Primers used for RNA analysis are listed in Table 3.

### Statistics

The Student *t* test was used for comparing mean ABR thresholds. Semi-quantitative data assessing ear pathology were analyzed by a chi-square test. A value of P<0.05 was considered significant.

## Supporting Information

Video S1 A *Chd7^Ome/+^* mouse showing circling behavior in Video S1.
(MP4)

Video S2 A Chd7Ome/+ mouse showing poor swimming ability (Video S2).
(MP4)

Video S3 A Chd7+/+ mouse showing normal swimming ability with his nose above water (Video S3).
(MP4)
